# Practical Points

**Published:** 1904-01

**Authors:** 


### PRACTICAL POINTS.

Very fine emery cloth such as jewelers use is found effective for cleaning cement spatulas and other instruments where a polished surface is desired.—*Review.*

I use a comparatively new clock spring cutting off small sections as needed, for matrices, and find them satisfactory.—*Register.*

If a gas has taken possession of the surface of gold, and meets with another and forms a salt that is not evaporated by the heat of annealing, the welding property of the gold is permanently lost.—*Headlight.*

A Useful Hemostatic.—Cotton soaked with oil of turpentine and pressed into the tooth socket after extraction will check the hemorrhage promptly.—*Medical Summary.*

Plaster of Paris, when it has incorporated with it an equal amount of powdered kaolin (Chinese clay), I have found it gives excellent results as material for models. The Chinese clay overcomes the changing disposition of paster and yields a smooth and strong model.—*Amer. Jour.*

Embalming Paste.—I have [illegible] ment box that contains four bottles, zinc, alum, thymol and glycerol. A speck of each of the first three named mixed with the glycerol makes a good embalming paste, if used fresh.—*Review.*

The pain causel by setting a crown or bridge may be alleviated almost instantly by the free application of campho-phenique upon the gum.—*Review.*

Sensitive Dentin.—Jarring the tooth with an automatic mallet, having a blunt-point plugger in the cavity, aids materially in inducing the penetration of fluids into the dentin.—*Headlight.*

Sensitive Dentin.—In cavities where the dentin is sensitive I take a pledget of cotton, thrust it into the spirit lamp and let it ignite, and while hot place it in the cavity and leave it there. I find that in many such cases I can cut sensitive dentin very much better after this treatment.—*Era.*

To remove Adhering Plaster from Plates, place the plate for a short time in water containig a small quantity of sulphate of postassium.—*Forum.*

The removal of green tartar from the teeth of little patients who will not tolerate the engine and pumice, can be readily effected by the application of tincture of iodin on a pledget of cotton, followed by ammonia in the same manner. This method is safe, painless, and effective.—*Brief.*

A tooth does not look as dark, as a matter of fact, when you place it in the mouth, as it does when you see it on the shade bar, and it is rather a good idea, where you have a finicky patient, to give him the impression, at least, that you let him have his way in the selection, then when you get ready for him, select such

